# Enhanced quality of nutrition services during antenatal care through interventions to improve maternal nutrition in Bangladesh, Burkina Faso, Ethiopia, and India

**DOI:** 10.7189/jogh.15.04054

**Published:** 2025-03-14

**Authors:** Phuong H Nguyen, Lan M Tran, Shivani Kachwaha, Tina Sanghvi, Zeba Mahmud, Maurice G Zafimanjaka, Tamirat Walissa, Sebanti Ghosh, Sunny S Kim

**Affiliations:** 1 Nutrition, Diets, and Health Unit, International Food Policy Research Institute (IFPRI), Washington D.C., USA; 2 Hubert Department of Global Health, Emory University, Atlanta, Georgia, USA,; 3 Program in Human Nutrition, Department of International Health, Johns Hopkins University, Baltimore, Maryland, USA; 4 FHI 360, Washington D.C., USA; 5 FHI 360, Dhaka, Bangladesh; 6 FHI 360, Ouagadougou, Burkina Faso; 7 FHI 360, Addis Ababa, Ethiopia; 8 FHI 360, New Delhi, India

## Abstract

**Background:**

Quality antenatal care (ANC) services are critical for maternal health and nutrition. Information on the quality of nutrition interventions during ANC is scarce in low- and middle-income countries. We examined the effects of intensified maternal nutrition interventions during ANC on service readiness, provision of care, and experience of care and assessed the inter-relationships between the dimensions of quality.

**Methods:**

We used data from impact evaluations of maternal nutrition interventions in Bangladesh, Burkina Faso, Ethiopia, and India. We calculated the quality of nutrition services during ANC using information from health facility assessments, health care provider interviews, ANC observations, and client exit interviews. We used structural equation models to examine relationships between the dimensions of quality.

**Results:**

Health facilities in all four countries had a high service readiness component in terms of basic amenities, equipment and supplies, medicines and commodities, and guidelines (mean (x̄) = 8–10 in Bangladesh and Burkina Faso, x̄ = 7–9 in Ethiopia, and x̄ = 6–8 in India). Scores for provision of care were low across the countries but higher in intervention compared to control areas in Bangladesh (5.2 *vs.* 2.9) and Burkina Faso (5.6 *vs.* 4.8), but not significantly different in Ethiopia (range = 4.7–5.0) and India (range = 2.6–3.5). For experience of care, client satisfaction scores were high and similar between intervention and control areas in all countries (range = 8.3–9.7), but client experience scores were lower with statistically significant differences observed only in Bangladesh (x̄ = 8.2 in intervention *vs*. x̄ = 7.1 in control areas). The interventions had significant direct effects on service readiness in Bangladesh (*β* = 0.07), Burkina Faso (*β* = 1.20), and Ethiopia (*β* = 1.0), on the provision of care in Bangladesh (*β* = 2.27), Burkina Faso (*β* = 1.27), and India (*β* = 0.96), and experience of care in Bangladesh (*β* = 0.21).

**Conclusions:**

In this study, we provided evidence on various dimensions of service quality that may be improved by interventions to strengthen nutrition services during ANC in diverse low- and middle-income countries.

Antenatal care (ANC) encompasses a range of essential health and nutrition services during pregnancy, including health promotion, diagnosis, disease treatment, and prevention. Adequate coverage and quality of ANC are critical for maternal and newborn health and nutrition outcomes, particularly in low- and middle-income countries (LMICs) where a high burden of maternal undernutrition and poor service quality have been long-standing concerns [1,2].

The World Health Organization (WHO) quality of care framework for maternal and newborn health care evaluates ANC quality on three dimensions: the health system (inputs and structure), the content of care (services or interventions), and the experience of care (communication, respect, and emotional support) [3–5]. While ANC coverage indicators such as the timing of the first ANC visit and the number and timing of ANC visits are well-documented [6], there is growing evidence highlighting the need to assess ANC quality better.

In recent years, the Demographic and Health Survey’s Service Provision Assessments [7] and WHO’s Service Availability and Readiness Assessment [8] surveys have been used to assess the quality of ANC in different contexts, capturing certain elements of the WHO quality of care framework [9–13]. However, these surveys focus on inputs and do not adequately capture the broad range of service provision and the experience of care [14]. Also, the variability in definitions and analytical approaches across studies complicate comparisons and limit our understanding of the relationships between different dimensions of ANC quality [14–16]. There is a need to understand the inter-relationships between the three dimensions of service readiness, provision of care, and experience of care.

Nutrition interventions are an essential component of ANC services. However, reporting on indicators related to nutrition interventions during ANC is often inconsistent and geographically variable [17,18]. Specifically, indicators for counselling on maternal nutrition and breastfeeding, iron and folic acid (IFA), and other micronutrient supplementation are limited. In addition, nutrition interventions such as counselling on adequate maternal nutrition and breastfeeding practices may not be a standardised service at every ANC visit or across different sites, making their measurement of quality during ANC challenging. Although evidence-based nutrition interventions have been scaled effectively in several countries [19,20], consistent measurement and evaluation of the quality of these services are lacking.

In four countries across Africa and South Asia – Bangladesh, Burkina Faso, Ethiopia, and India – the Alive & Thrive (A&T) initiative worked with national governments and local collaborators to strengthen the delivery of maternal nutrition interventions within existing ANC platforms [21–24]. Impact evaluation results showed improvements in maternal diet and early breastfeeding practices [21–24], but the quality of nutrition services during ANC have not yet been assessed. The similar designs and measures across these studies provided an opportunity to examine this issue in multiple sites and fill an evidence gap.

This study is a secondary data analysis using impact evaluation datasets of maternal nutrition studies in four countries with diverse socio-economic, cultural, and health system contexts – Bangladesh, Burkina Faso, Ethiopia, and India. Our objectives were to elucidate the differences in service readiness, provision of care, and experience of care of nutrition interventions during ANC between intervention and control areas across country settings and to examine the relationships between the different dimensions of quality of care. We hypothesised that areas with interventions will demonstrate higher service quality than control areas. Additionally, we expected that the dimensions of service readiness, provision of care, and care experience will be closely interconnected, reflecting a comprehensive improvement in the overall quality of care.

## METHODS

### A&T intervention studies and data sources

Impact evaluations using a quasi-experimental design in Bangladesh (2019–21) and a cluster-randomised design in India (2017–19), Burkina Faso (2019–21), and Ethiopia (2019–21) were conducted by the International Food Policy Research Institute (IFPRI) to evaluate the impact of strengthening nutrition interventions during ANCs on maternal nutrition practices. While the platform for ANC service delivery was non-governmental/governmental health facilities in Bangladesh, Burkina Faso and Ethiopia, the platform in India was outreach/community-based.

Details about the study design, data collection methods, and results of the impact evaluations have been reported elsewhere [21–24]. Briefly, the quasi-experimental design in Bangladesh included non-random assignment of health facilities to intervention and control groups by propensity score matching to select controls based on potential confounding variables. In the cluster-randomized designs, intervention and control groups were randomly assigned through multi-level sampling using pair-matched randomisation or stratified random allocation. Data were collected through structured questionnaires via face-to-face interviews.

For secondary data analysis in this study, we used multiple datasets collected at the endline across the four countries. The data sources included health facility assessments (n = 16 in Bangladesh, n = 80 in Burkina Faso, n = 30 in Ethiopia, n = 75 in India), interviews with health care providers (n = 72 in Bangladesh, n = 79 in Burkina Faso, n = 30 in Ethiopia, n = 127 in India), observations of ANC including nutrition counselling (n = 1215 in Bangladesh, n = 158 in Burkina Faso, n = 60 in Ethiopia, n = 212 in India), and client exit interviews (n = 1215 in Bangladesh, n = 158 in Burkina Faso, n = 60 in Ethiopia, n = 212 in India). The health facility assessments used a checklist developed in consultation with program implementation partners to assess the condition of the facilities, amenities, services, and human resources. Healthcare provider interviews used a structured questionnaire to collect information about nutrition knowledge, job motivation, and supervision of ANC services. For the ANC observations, a checklist based on the intervention protocol in each country was used to record assessments, activities, messages, and interpersonal communication skills during ANC visits. After each ANC observation, a client exit interview was conducted to gather information about client experience and satisfaction with the services received. Data was collected by trained enumerators, and fieldwork was supervised by field supervisors and IFPRI researchers.

### Conceptualising quality of nutrition services during ANC

The conceptual framework for this study (Figure S1 in the **Online Supplementary Document**) focuses on three key dimensions of quality of care, particularly for ANC. First, service readiness refers to potential inputs foundational for quality services. It is measured by the availability of both material and human resources, including basic amenities, equipment, medicines and commodities, diagnostics, guidelines, trained staff, and supervision. Further, provision of care refers to the quality of service delivery by providers to clients, categorised into assessment (physical exams, routine tests, weight monitoring, and weight gain counselling), intervention (counselling content and prescription/provision of vitamins, minerals, and essential medicines), and documentation (medical records). Experience of care consists of client experience and client satisfaction based on effective communication by the service provider, client expectations, respect and preservation of dignity, and client access to emotional and social support of their choice.

We matched items in our data sources to each dimension of quality of care and their corresponding components, as shown in the conceptual framework. We adapted the definitions and items for quality of care metrics from Latoff et al. and King et al. [4,25]. Following a systematic process of review and expert consultation, King et al. proposed 40 items for inclusion across the dimensions of service readiness, provision of care, and experience of care for overall ANC quality [25]. The maternal nutrition interventions included assessment and treatment of anaemia, blood glucose testing, calcium supplementation, iron and folic acid supplementation, nutrition counselling and education, intermittent preventative treatment for malaria, and deworming [25,26]. We mapped each of the 40 items to our study data and included additional items under the provision of care related to counselling on maternal nutrition and breastfeeding that were relevant and available in our study but not included in King et al. We mostly included items available across the four-country datasets to enable cross-country comparisons while considering the national policies and guidelines.

### Quality of care indices

For service readiness, we created five components (basic amenities, equipment and supplies, medicines and commodities, diagnostics, guidelines, and information, education and communication (IEC) materials) from items in the health facility assessments. We created staff training and supervision components from questions in the health care provider interviews. We generated each item as a binary variable, with ‘1’ representing the availability and ‘0’ representing the absence of the item in the facilities. Items in staff training and supervision included any ANC/maternal nutrition training or supervision conducted, topics covered in the training, and actions conducted during supervision visits. We added all the available items in each component to create the score of the component. The composite service readiness dimension score was the sum of the seven components.

We employed a similar method to calculated scores of the provision of care and experience of care dimensions, using items in the ANC observations and client exit interviews. Due to different country priorities, the types of interventions varied slightly. Bangladesh and India, for example, included calcium supplementation, while deworming and malaria interventions were included in Burkina Faso and Ethiopia. Also, actions and messages provided varied across countries, resulting in differences in the raw scores of the components. Therefore, we standardised the component and dimension scores into a 10-point scale to facilitate cross-country comparisons.

### Statistical analysis

Descriptive statistics were used to report health care providers’ and clients’ characteristics, as well as percentages or means (x̄) and standard deviation of the components and the dimensions of quality. We assessed the statistical differences in the scores of the components and the dimensions between the intervention and control areas using two-sided *t* tests to detect potential unexpected effects.

We employed structural equation models to evaluate the relationships between the dimensions of the quality of care indices and to quantify the size and direction of the intervention’s influence on the experience of care through direct or indirect pathways via service readiness and provision of care. The models assumed that the intervention could affect service readiness and provision of care, which in turn could affect the experience of care. We adjusted direct and indirect associations between the dimensions for the A&T treatment groups. We assessed model fit based on the model χ^2^ (<0.05), comparative fit index value (>0.95), and root mean square error of approximation value (<0.001) [27]. The statistical significance of differences between intervention and control areas was presented as *P*-value <0.05. We analysed data using Stata, version 17 (StataCorp, College Station, Texas, USA).

## RESULTS

### Sample characteristics

Healthcare providers ranged in age from x̄ = 25 years in Ethiopia and x̄ = 45 in India. The majority were female across the countries. The percentage of health care providers with college/university or higher education varied by country (65–81% in Bangladesh, 8–13% in Burkina Faso, 20–60% in Ethiopia, and 42–59% in India). The work duration ranged from x̄ = 3–7 years in Bangladesh, Burkina Faso, and Ethiopia to x̄ = 15–18 years in India (**Table 1**). On average, clients at ANC visits were about 25 years old and in their second trimester of pregnancy across the countries (**Table 2**).

**Table 1:** Healthcare provider characteristics in Bangladesh, Burkina Faso, Ethiopia, and India*

	Bangladesh		Burkina Faso		Ethiopia		India	
**Healthcare providers characteristics**	**Intervention (n = 41)**	**Control (n = 31)**	**Intervention (n = 40)**	**Control (n = 39)**	**Intervention (n = 15)**	**Control (n = 15)**	**Intervention (n = 58)**	**Control (n = 69)**
Age in years, x̄ (SD)	34.7 (8.1)	38.8 (8.2)	36.3 (5)	35.5 (5.2)	26.1 (3.5)	25.3 (2)	45.2 (11.5)	41.9 (11.6)
Female	100.0	100.0	77.5	76.9	66.7	53.3	100.0	100.0
Education								
*Completed secondary/vocational school*	19.5	35.5	87.5	92.3	40.0	80.0	58.6	42.0
*College/university or higher*	80.5	64.5	12.5	7.7	60.0	20.0	41.4	58.0
Working duration in years, x̄ (SD)	4.7 (3.6)	7.1 (5.9)	7.1 (5.1)	5.8 (5.2)	4.2 (3.3)	2.9 (1.6)	18.4 (11.6)	14.9 (12)
*<2*	24.4	22.6	15.0	25.6	26.7	20.0	1.7	11.6
*2–5*	34.1	19.4	22.5	23.1	46.7	66.7	13.8	14.5
*5–10*	31.7	32.3	30.0	25.6	26.7	13.3	17.2	18.8
*≥10 y*	9.8	25.8	32.5				67.2	55.1

SD – standard deviation, x̄ – mean

*Presented as % unless specified otherwise.

**Table 2:** Client characteristics in Bangladesh, Burkina Faso, Ethiopia, and India*

	Bangladesh		Burkina Faso		Ethiopia		India	
**Clients’ characteristics**	**Intervention n = 720**	**Control n = 495**	**Intervention n = 80**	**Control n = 87**	**Intervention n = 30**	**Control n = 30**	**Intervention n = 104**	**Control n = 108**
Age in years, x̄ (SD)	24.6 (5.0)	24.2 (5.3)	26.4 (6.2)	27.3 (6.2)	28.3 (5.8)	25.2 (5.4)	25.0 (4.0)	25.2 (3.9)
Duration of pregnancy in weeks, x̄ (SD)	20.3 (8.5)	20.0 (7.9)	26.2 (8.5)	25.0 (7.8)	26.1 (9.7)	24.2 (10.0)	22.3 (7.3)	20.9 (7.7)
ANC visit number for the current pregnancy								
*1*	42.4	39.4	28.8	34.6	23.3	23.3		
*2*	26.4	26.4	11.3	19.2	33.3	36.7		
*3*	13.1	17.2	12.5	29.5	13.3	16.7		
*≥4*	18.2	17.0	47.5	16.7	30.0	23.3		
Number of pregnancies								
*1*			17.5	12.8	16.7	23.3	39.4	34.3
*2*			82.5	87.2†	83.3	76.7†	26.9	33.3
*3*							17.3	21.3
*≥4*							16.4	11.1

ANC – antenatal care, SD – standard deviation, x̄ – mean

*Presented as % unless specified otherwise.

†≥2 pregnancies in Burkina Faso and Ethiopia.

### Service readiness

**Figure 1:**
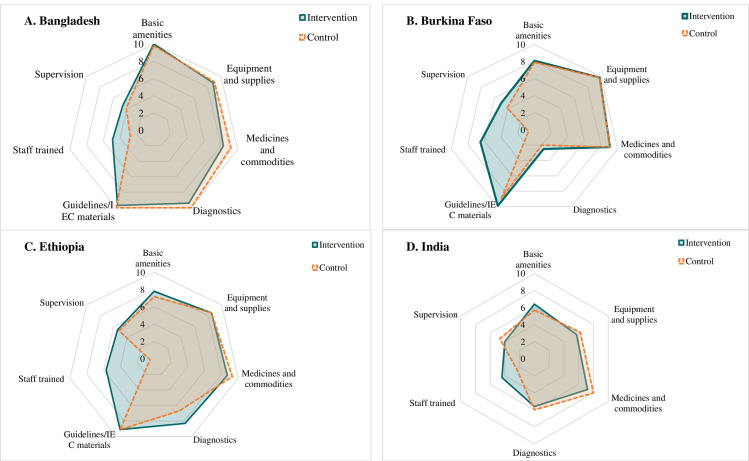
Service readiness components. **Panel A.** Bangladesh. **Panel B.** Burkina Faso. **Panel C.** Ethiopia. **Panel D.** India.

Overall, health facilities across countries had high service readiness in both the intervention and control areas in terms of basic amenities, equipment and supplies, medicines, and commodities, and guidelines and IEC (**Figure 1**). For these components, the x̄ scores out of 10 ranged from x̄ = 8–10 in Bangladesh and Burkina Faso, x̄ = 7–9 in Ethiopia, and x̄ = 6–8 in India (Table S1 in the **Online Supplementary Document**). Diagnostics were also high for three countries (x̄ = 6–10) but low for Burkina Faso (x̄ = 2–2.5). For training, the x̄ scores were significantly higher in the intervention (x̄ = 4–7) than in control areas (x̄ = 1–3) across all countries. Supportive supervision had similar low x̄ scores in intervention and control areas across the countries (x̄ = 4–5). The x̄ composite service readiness scores out of 10 were similar in Bangladesh (x̄ = 8) and India (x̄ = 6) but higher in intervention *vs.* control in Burkina Faso (x̄ = 7.3 *vs.* x̄ = 6.1) and Ethiopia (x̄ = 7.7 *vs.* x̄ = 6.6). Details on individual components of service readiness are provided in Table S2 in the **Online Supplementary Document**.

### Provision of care

**Figure 2:**
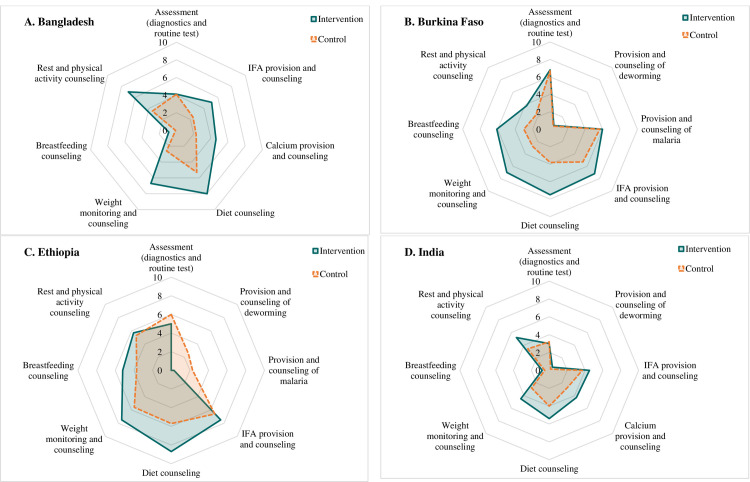
Provision of care components. **Panel A.** Bangladesh. **Panel B.** Burkina Faso. **Panel C.** Ethiopia. **Panel D.** India.

Provision of care was low overall, with few variations in the performance of different interventions across countries (**Figure 2**; Table S1 in the **Online Supplementary Document**). Scores on assessment were similar for intervention and control areas, with the highest scores in Burkina Faso (x̄ = 7) and the lowest in India (x̄ = 3). Provision of and counselling on deworming were unavailable in Bangladesh and particularly low in the other three countries (x̄ = 0–1). Provision of and counselling on malaria were available only in Burkina Faso and Ethiopia and similar in the intervention compared to control areas. Scores on iron and folic acid provision and counselling were higher in the intervention than in control areas in Bangladesh (x̄ = 5.1 *vs.* x̄ = 2.4), and Burkina Faso (x̄ = 7.2 *vs.* x̄ = 5.3), but the differences between treatment areas were not significant in Ethiopia and India. Similar results were observed for calcium supplement provision and counselling. Counselling about adequate diet and weight monitoring/weight gain counselling had higher scores in the intervention (x̄ = 5–8) compared to control areas (x̄ = 3–5) across all the countries. Breastfeeding counselling scores were particularly low for Bangladesh and India (x̄ = 0–1) but higher in Burkina Faso and Ethiopia.

The composite scores for provision of care were low across the countries but higher in the intervention compared to control areas in Bangladesh (x̄ = 5.2 *vs.* x̄ = 2.9) and Burkina Faso (x̄ = 5.6 *vs.* x̄ = 4.8), but not significantly different in Ethiopia (x̄ = 4.7–5), and India (x̄ = 2.6–3.5) (Table S1 in the **Online Supplementary Document**). Details on individual components of provision of care are provided in Table S3 in the **Online Supplementary Document**.

### Experience of care

Client satisfaction scores were high and similar between intervention and control areas in all countries (x̄ = 8.3–9.7 in Bangladesh, Burkina Faso, and Ethiopia) (**Table 3**). Client experience scores were lower than client satisfaction scores, with statistically significant differences observed only in Bangladesh (x̄ = 8.2 in intervention *vs.* x̄ = 7.1 in control areas). The client experience score was much lower in India than in other countries, resulting in a low overall experience of care score (x̄ = 4.5–4.9 in control and intervention areas, respectively). Description of items used to calculate the experience of care are presented in Table S4 in the **Online Supplementary Document**.

**Table 3:** Experience of care components in Bangladesh, Burkina Faso, Ethiopia, and India*

	Bangladesh		Burkina Faso		Ethiopia		India	
**Clients’ experience of care**	**Intervention n = 720**	**Control n = 495**	**Intervention n = 80**	**Control n = 78**	**Intervention n = 30**	**Control n = 30**	**Intervention n = 104**	**Control n = 108**
Client satisfaction	9.7 (0.8)	9.7 (1.0)	9.7 (0.9)	9.6 (1.2)	8.8 (2.7)	8.3 (3.0)		
Client experience	8.2 (1.1)	7.1 (1.1)	9.3 (1.3)	8.9 (1.1)	7.8 (2.1)	7.9 (1.0)	4.9 (2.5)	4.5 (2.1)
Experience of care score†	9.0 (0.7)	8.4 (0.8)	9.5 (0.9)	9.2 (0.8)	8.3 (2.2)	8.1 (1.5)	4.9 (2.5)	4.5 (2.1)

*Presented as x̄ (SD) unless specified otherwise.

†Range 0–10.

### Relationships between different dimensions of quality of care

**Figure 3:**
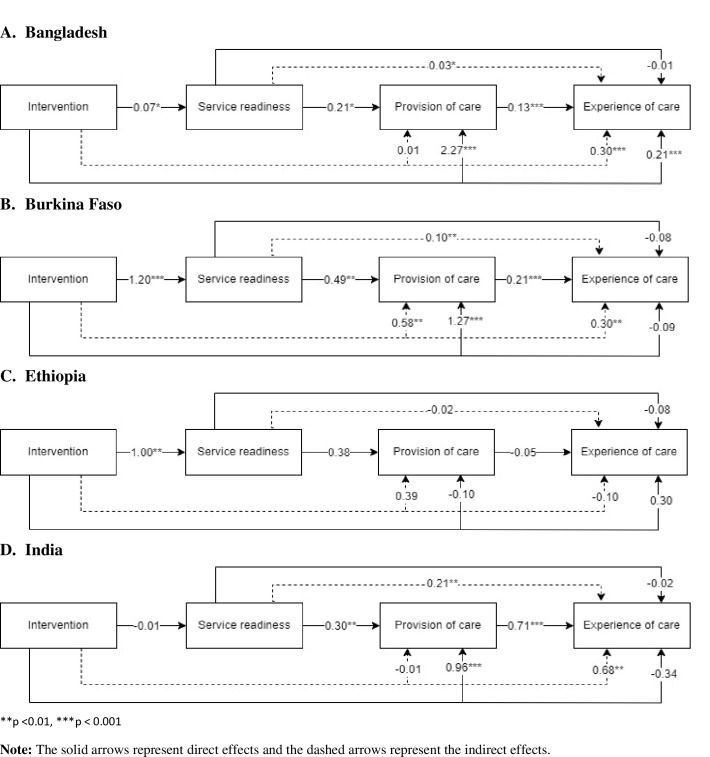
Relationships between different dimensions of quality of care. **Panel A.** Bangladesh. **Panel B.** Burkina Faso. **Panel C.** Ethiopia. **Panel D.** India.

Relationships between the dimensions of quality of care are presented in **Figure 3**. Higher service readiness was directly associated with higher provision of care (*β* = 0.21 for Bangladesh, *β* = 0.49 for Burkina Faso, *β* = 0.38 for Ethiopia, and *β* = 0.30 for India). Provision of care, in turn, was significantly associated with experience of care in Bangladesh (*β* = 0.13), Burkina Faso (*β* = 0.20), and India (*β* = 0.71), while no relationship was observed for Ethiopia. The indirect paths between service readiness and experience of care mediated by the provision of care were significant for Bangladesh (*β* = 0.03), Burkina Faso (*β* = 0.10), and India (*β* = 0.21). The maternal nutrition interventions were directly associated with higher scores of service readiness (*β* = 0.07 in Bangladesh, *β* = 1.20 in Burkina Faso, and *β* = 1.0 in Ethiopia), provision of care scores (*β* = 2.27 in Bangladesh, *β* = 1.27 in Burkina Faso, and *β* = 0.96 India), and experience of care in Bangladesh. The interventions were also indirectly associated with the experience of care through both service readiness and provision of care in Bangladesh (*β* = 0.30), Burkina Faso (*β* = 0.30), and India (*β* = 0.68).

## DISCUSSION

In this study, we provided evidence on the quality of care of nutrition services during ANC in diverse LMIC contexts and the association of integrated maternal nutrition interventions with quality indicators. We identified which aspects of service readiness, provision of care, and care experience were influenced by the interventions by examining the differences between intervention and control areas and across countries. The magnitude of the differences may be explained by the design and implementation of the interventions and the local context of existing health systems. Another key finding from our study is on the inter-relationships between the dimensions of quality of care, which highlight the direct relationships between the dimensions and the mediation of provision of care between service readiness and experience of care. We also observed that the interventions were directly and indirectly associated with quality of care across all the countries.

Service readiness scores in the four countries in our study were moderate to high, similar to findings on ANC quality from other studies [11,28]. Service readiness for nutrition services during ANC in our study were significantly higher in the intervention compared to control areas in Burkina Faso and Ethiopia, but the differences were marginal in Bangladesh and India. The main component that contributed to higher scores in the intervention area was the training of service providers, which was a major input of the interventions across the countries. While supportive supervision and IEC materials were also parts of the interventions by design, the scores of these components remained similar in both intervention and control areas, indicating their inadequate intensity above the control and/or potential spillover in the control areas. Across the countries, service readiness components were lowest in India, which may be due to differences in the intervention setting. The intervention model in India was primarily implemented through community-based activities in which basic amenities, supplies, and diagnostics are limited, whereas the interventions in the other three countries were conducted at primary health care facilities with higher levels of services. Findings from previous studies consistently reported the variation in service readiness by geographic regions and types of health facilities, indicating the gaps in the structural readiness of health facilities to provide quality ANC services [10,11,29]. Furthermore, training coverage was low even in intervention areas, which may be due to training being provided for only one or two health care workers who were mainly responsible for the ANC/maternal and child health services in each facility, while the assessments were conducted for several health care workers in the facilities.

For the provision of care, the overall scores were low to moderate, but we observed considerable variation across countries, service types, and treatment groups. Compared to control areas, intervention areas had higher scores for the provision of most nutrition services across the countries, although the differences were statistically significant in Bangladesh and Burkina Faso only. The small sample size in Ethiopia (n = 60) likely influenced the power to detect the difference in this context. In India, although the sample size was larger, the differences between intervention and control areas were too small to achieve statistical significance. Variations were observed by type of service, with the highest scores for diet counselling and the lowest scores for breastfeeding counselling and deworming. While focusing on an adequate diet to improve nutrition during pregnancy is crucial for both mother and newborn health, it is also essential to prioritise breastfeeding counselling in ANC to improve early breastfeeding practices during the postpartum period, which has proven benefits for mothers and infants [30]. The low provision of care and the variation across contexts may be explained by multiple factors, including the lack of specificity in national guidelines and protocols, supply-chain bottlenecks for micronutrient supplements, inadequate supervision, and low provider knowledge and skills [20]. Results from other studies also emphasised extremely wide variations in the provision of care scores across countries (35–85% reported in one analysis) and by intervention type [12,13,28].

Experience of care components was high and similar between treatment groups in Bangladesh, Burkina Faso, and Ethiopia. In India, the experience of care was low and similar between intervention and control areas. Lower scores in India may be explained by lower provision of care described above, as well as limited data availability capturing experience of care. Few studies have reported on clients’ experience of caregiven the limited availability of items within the Demographic and Health Survey’s Service Provision Assessments and WHO’s Service Availability and Readiness Assessment questionnaires that have been used to measure ANC quality of care. Among studies that reported on client experience in sub-Saharan Africa, lower experience scores were attributed to longer wait times at health facilities, cleanliness of the facility, and availability of medications [9,13].

Several inter-relationships were observed between the three dimensions of quality of care for nutrition interventions during ANC. The interventions had direct effects on service readiness in Bangladesh, Burkina Faso, and Ethiopia; on provision of care in Bangladesh, Burkina Faso, and India; and on experience of care in Bangladesh only. In addition, indirect intervention effects were observed for the client’s experience of care through the provision of care in Burkina Faso and India. The lack of direct intervention effects on service readiness in India could likely be explained by the community-based intervention model, which may not have resulted in changes to the health facility-related components. Service readiness had direct effects on the provision of care, and provision of care had direct effects on the experience of care in Bangladesh, Burkina Faso, and India. Provision of care was an important mediator between service readiness and experience of care in Bangladesh, Burkina Faso, and India. There is inconsistent evidence on associations between the dimensions of ANC quality from previous studies. For example, one analysis across five LMICs in South Asia and Sub-Saharan Africa found important associations between service readiness and provision of care [31]. On the other hand, another study found that the infrastructure of health facilities did not translate to improved provision or client satisfaction [13], while another study found that infrastructure was not associated with the provision of care in 8 Sub-Saharan African countries [12]. While our results contribute to identifying these key relationships, further research is needed for documenting generalisability by assessing consistency across studies.

There are several strengths and limitations to our study. To our knowledge, this is the first study to examine the quality of care of nutrition interventions during ANC in LMICs. We used multiple data sources at different levels (health facility, health care staff, and observations of service delivery) across four different country settings. Quality of care for nutrition interventions was constructed based on an established conceptual framework for overall ANC quality, with similar questionnaires and standard methods which allowed comparisons across countries. Data collection methods were tailored for the intended purpose of evaluating the A&T intervention models, thus quality measures focused primarily on nutrition interventions. The structural equation models quantified the extent to which A&T interventions, directly and indirectly, influenced each dimension of quality of care in different country contexts. However, the analysis relies on cross-sectional data, which limits the assessment of relationships overtime on the quality-of-care dimensions and might influence the estimated associations. In addition, the relatively small sample size of certain data sets (for example, in Ethiopia) may limit the statistical power to detect differences between intervention and control areas. Potential biases inherent in the secondary data analyses, such as reporting bias, recall bias, measurement error, or incomplete information, were addressed by employing rigorous data collection methods and standardising evaluation tools across countries.

## CONCLUSIONS

This study contributes insights into the gaps between implementation and service quality across multiple and diverse LMICs contexts. Our findings highlight the potential for health systems to strengthen interventions focused on nutrition to improve the quality of nutrition interventions during ANC, particularly for the provision of care and experience of care, and the key inter-relationships between different dimensions of quality of ANC. We recommend that future research to improve nutrition services should consider potential supply and demand-side factors that may influence quality of care such as capacity and supervision of health care providers and client characteristics.

## Additional material


Online Supplementary Document
