# Supplementary material for: Enhanced quality of nutrition services during antenatal care through interventions to improve maternal nutrition in Bangladesh, Burkina Faso, Ethiopia, and India
Source: J Glob Health. 2025 Mar 14;15:04054. doi: 10.7189/jogh.15.04054 (PMC11906204; doi:10.7189/jogh.15.04054)
Supplement: Online Supplementary Document [file jogh-15-04054-s001.pdf]

Supplement to: Nguyen PH, Tran LM, Kachwaha S, Sanghvi T, Mahmud Z, Zafimanjaka MG, Walissa T, Ghosh S, Kim SS. Enhanced quality of nutrition services during antenatal care through interventions to improve maternal nutrition in Bangladesh, Burkina Faso, Ethiopia, and India. J Glob Health. 2025;15:04054.

Figure S1: Conceptual framework

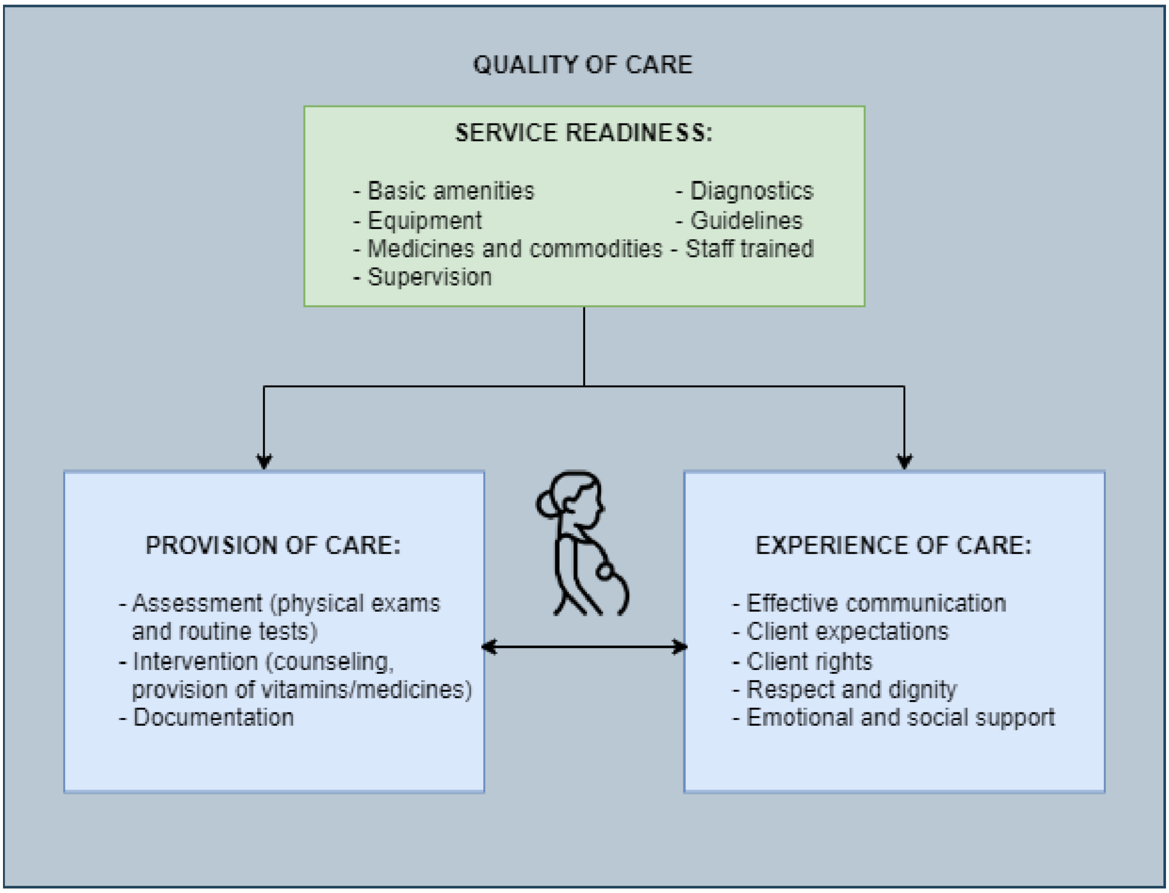

Table S1: ANC quality characteristics in Bangladesh, Burkina Faso, Ethiopia, and India[1]

	BANGLADESH		BURKINA FASO		ETHIOPIA		INDIA	
	Intervention	Control	Intervention	Control	Intervention	Control	Intervention	Control
	Mean (SD)	Mean (SD)	Mean (SD)	Mean (SD)	Mean (SD)	Mean (SD)	Mean (SD)	Mean (SD)
**SERVICE READINESS**	**N=8**	**N=8**	**N=40**	**N=40**	**N=15**	**N=15**	**N= 35**	**N= 40**
Basic amenities	10 (0)	9.8 (0.6)	8.1 (1.3)	7.9 (1.3)	7.8 (1.9)	7.2 (2)	6.4 (2.4)	5.7 (2.5)
Equipment and supplies	8.8 (1.5)	9 (1.1)	9.8 (0.8)	9.8 (0.5)	8.5 (1.6)	8.5 (1.2)	5.7 (2.8)	6.2 (2.0)
Medicines and commodities	8.3 (1.8)	9.2 (1.5)	9.1 (1.7)	9.1 (1.8)	8.7 (2.1)	9.3 (1.4)	7.2 (2.7)	8.0 (2.4)
Diagnostics	9.4 (1.8)	10 (0)	2.5 (2.5)	2 (2.5)	8.3 (2.4)	6.7 (3.6)	5.6 (3.8)	6.0 (3.4)
Guidelines/IEC materials	9.7 (0.9)	10 (0)	9.9 (0.5)	8.9 (2.2)*	9.1 (2)	9.1 (1.5)	--	--
Staff trained	4.9 (2.2)	2.8 (1.7)*	6.5 (3.6)	0.7 (2)***	5.7 (3.9)	0.5 (1.8)***	4.4 (3.2)	2.4 (3.1)**
Supervision	4.6 (0.7)	4.1 (1.0)	5.0 (2.0)	4.1 (2.2)$^{+}$	5.4 (2.3)	5.2 (2.2)	4.0 (1.9)	4.7 (2.1)$^{+}$
Service readiness score	***8.0 (0.8)***	***7.8 (05)***	***7.3 (0.9)***	***6.1 (0.8)****	***7.7 (1.6)***	***6.6 (0.9)****	***5.6 (1.4)***	***5.5 (1.1)***
**PROVISION OF CARE**	**N=720**	**N=495**	**N=80**	**N=78**	**N=30**	**N=30**	**N= 104**	**N= 108**
Assessment (diagnostics and routine test)	4.1 (2)	4.1 (2.3)	6.8 (1.3)	6.7 (0.9)	5 (2.7)	6 (2.2)	3 (2)	3.2 (2.1)
Provision and counseling of deworming medicines	--	--	0.6 (2.2)	0.5 (2.1)	0 (0)	2.6 (4)*	0.5 (2.1)	0.2 (1.4)
Provision and counseling of malaria prevention/treatment	--	--	6 (3.2)	5.7 (2.8)	0.3 (0.8)	2.3 (3.6)$^{+}$	--	--
IFA provision and counseling	5.1 (2.6)	2.4 (1.7)***	7.2 (1.9)	5.3 (2)***	7.5 (2)	6.6 (3.3)	4.5 (3.1)	3.7 (2.6)
Calcium provision and counseling	4.6 (2.9)	2.3 (1.9)**	--	--	--	--	4.3 (3.4)	2.8 (2.9)$^{+}$
Diet counseling	8 (3.4)	5.3 (3.3)*	7.5 (2.1)	3.8 (3.5)***	8.7 (2.6)	5.7 (3.5)**	5.4 (3.6)	4 (3.4)$^{+}$
Weight monitoring and weight gain counseling	6.7 (3.8)	2.6 (3.2)***	7 (3.4)	2.8 (3.2)***	7.5 (3.8)	5.6 (4.1)	4.5 (4.1)	2.8 (3.6)$^{+}$
Breastfeeding counseling	0.9 (2.4)	0.1 (0.9)***	6.1 (3.2)	3 (3.4)***	5.2 (4.3)	3.7 (4.7)	0.8 (2.4)	0.5 (1.8)
Rest and physical activity counseling	7 (3.2)	3.5 (3.1)***	3.8 (3.6)	2.3 (2.9)*	5.7 (3.2)	5.3 (3.3)	5.2 (4.6)	3.4 (4.3)$^{+}$
Provision of care score	**5.2 (2)**	**2.9 (1.5)*****	**5.6 (1.7)**	**3.8 (1.8)*****	**5.0 (1.5)**	**4.7 (2.8)**	**3.5 (2.1)**	**2.6 (1.7)**$^{+}$
**EXPERIENCE OF CARE**	**N=720**	**N=495**	**N=80**	**N=78**	**N=30**	**N=30**	**N= 104**	**N= 108**
Client satisfaction	9.7 (0.8)	9.7 (1)	9.7 (0.9)	9.6 (1.2)	8.8 (2.7)	8.3 (3)	--	--
Client experience	8.2 (1.1)	7.1 (1.1)***	9.3 (1.3)	8.9 (1.1)	7.8 (2.1)	7.9 (1)	4.9 (2.5)	4.5 (2.1)
Experience of care score	**9 (0.7)**	**8.4 (0.8)****	**9.5 (0.9)**	**9.2 (0.8)**	**8.3 (2.2)**	**8.1 (1.5)**	**4.9 (2.5)**	**4.5 (2.1)**

[1]Possible range: 0 – 10

Note: Student t-tests were conducted to compare the difference between intensive and non-intensive areas; p-values report the significant difference accounting for geographic clustering. $^{+}p<0.10$, $*p < 0.05$, $**p < 0.01$, $***p < 0.001$. IEC- Information education and communication, IFA- Iron and folic acid

Table S2: Service readiness components in Bangladesh, Burkina Faso, Ethiopia, and India

	BANGLADESH		BURKINA FASO		ETHIOPIA		INDIA	
	Intervention N= 8	Control N=8	Intervention N=40	Control N=40	Intervention N=15	Control N=15	Intervention N= 35	Control N= 40
	%/ Mean (SD)	%/ Mean (SD)	%/ Mean (SD)	%/ Mean (SD)	%/ Mean (SD)	%/ Mean (SD)	%/ Mean (SD)	%/ Mean (SD)
**Basic amenities**								
Clean environment	100	87.5	72.5	82.5	66.7	46.7		
Power supply	100	100	100	100	100	100	25.7	27.5
Water supply	100	100	95	87.5	73.3	66.7	80	77.5
Toilet facility	100	100	95	97.5	100	100	71.4	65
Handwashing facility	100	100	100	92.5	66.7	46.7	85.7	80
Counseling room	100	100	25	12.5	60	73.3	57.1	35
Basic amenities score[1]	**10 (0)**	**9.8 (0.6)**	**8.1 (1.3)**	**7.9 (1.3)**	**7.8 (1.9)**	**7.2 (2)**	**6.4 (2.4)**	**5.7 (2.5)**
**Equipment and supplies**								
Blood pressure instrument	100	100	100	100	100	100	62.9	67.5
Stadiometer or height rod	87.5	100	97.5	100	93.3	86.7	17.1	12.5
MUAC tape	50	100	100	100	86.7	100		
Adult weighing scale	100	100	100	100	100	100	80	87.5
Handwashing soap	100	50	92.5	92.5	46.7	40	68.6	80
Equipment and supplies score[1]	**8.8 (1.5)**	**9 (1.1)**	**9.8 (0.8)**	**9.8 (0.5)**	**8.5 (1.6)**	**8.5 (1.2)**	**5.7 (2.8)**	**6.2 (2)**
**Medicines and commodities**								
Iron-folic acid tablets	100	100	100	97.5	93.3	93.3	91.4	100.0
Calcium supplements	100	100					85.7	75.0
Albendazole/Mebendazole	50	75	75	80	66.7	86.7	40.0	65.0
Sulfadoxine-pyrimethamine for IPTp			97.5	95	100	100		
Medicines and commodities score[1]	**8.3 (1.8)**	**9.2 (1.5)**	**9.1 (1.7)**	**9.1 (1.8)**	**8.7 (2.1)**	**9.3 (1.4)**	**7.2 (2.7)**	**8 (2.4)**
**Diagnostics**								
Hemoglobin levels	87.5	100	0	0	80	46.7	77.1	85.0
Urine testing kit	100	100	50	40	86.7	86.7	34.3	35.0
Diagnostics score	**9.4 (1.8)**	**10 (0)**	**2.5 (2.5)**	**2 (2.5)**	**8.3 (2.4)**	**6.7 (3.6)**	**5.6 (3.8)**	**6 (3.4)**
**Guidelines/IEC materials**								
Guidelines for ANC	100	100	97.5	82.5	80	100		
Guidelines for IYCF	87.5	100						
IEC materials on maternal nutrition	100	100	100	87.5	100	93.3		
IEC materials on IYCF	100	100	100	97.5	93.3	80		
Guidelines/IEC materials score	**9.7 (0.9)**	**10 (0)**	**9.9 (0.5)**	**8.9 (2.2)**	**9.1 (2)**	**9.1 (1.5)**		
**Staff trained**								

	BANGLADESH		BURKINA FASO		ETHIOPIA		INDIA	
	**Intervention**	**Control**	**Intervention**	**Control**	**Intervention**	**Control**	**Intervention**	**Control**
Staff trained in the last 1 year in ANC/maternal nutrition	55.8	43.3	80	10.3	73.3	6.7	74.3	42.5
Topics covered in training								
Early registration and number of ANC visits			57.5	7.7	33.3	0	25.7	12.5
Importance of maternal nutrition	52.2	35	77.5	10.3	66.7	6.7	37.1	30
Weight monitoring and weight gain	43.1	5.6	62.5	2.6	60	0	28.6	12.5
Diet intake during pregnancy	51.2	32.5	47.5	5.1	53.3	6.7	54.3	32.5
IFA and calcium supplementation	36.6	10.6	50	0	73.3	6.7	45.7	25
Breastfeeding	53.7	43.3	77.5	10.3	40	6.7	42.9	15
***Staff trained score*[1]**	**4.9 (2.2)**	**2.8 (1.7)**	**6.5 (3.6)**	**0.7 (2)**	**5.7 (3.9)**	**0.5 (1.8)**	**4.4 (3.2)**	**2.4 (3.1)**
**Supervision**								
Contacted with direct supervisor in the last 1 month	94.8	91.2	100	97.4	93.3	86.7	88.6	92.5
Received supervisory visit in last 1 month	95.4	100	65.0	69.2	93.3	86.7	48.6	70
Actions conducted in the last supervisory visit								
Check registers and records	78.4	72.3	62.5	51.3	66.7	86.7	62.9	60
Check/observe antenatal care counselling	28.1	3.1	55.0	43.6	66.7	53.3	28.6	27.5
Gave information about maternal nutrition or breastfeeding	14.9	3.1	52.5	23.1	46.7	40.0	11.4	30
Provide feedback	39.2	39.0	27.5	23.1	26.7	20.0	14.3	12.5
Provide job aids and other materials	9.4	5.6	20.0	10.3	20.0	33.3		
Planning or scheduling activities	10.3	10.0	17.5	12.8	20.0	6.7	25.7	37.5
***Supervision score*[1]**	***4.6 (0.7)***	***4.1 (1.0)***	***5.0 (2.0)***	***4.1 (2.2)***	***5.4 (2.3)***	***5.2 (2.2)***	***4 (1.9)***	***4.7 (2.1)***

[1]Possible range: 0 – 10

Note: MUAC- Mid-upper arm circumference, IPTp- Intermittent preventive treatment of malaria during pregnancy, IEC- Information education and communication, ANC- Antenatal care, IYCF- Infant and young child feeding, IFA- Iron and folic acid

Table S3: Provision of care components in Bangladesh, Burkina Faso, Ethiopia, and India

	BANGLADESH		BURKINA FASO		ETHIOPIA		INDIA	
	Intervention N=720	Control N=495	Intervention N=80	Control N=78	Intervention N=30	Control N=30	Intervention N= 104	Control N= 108
	%/ Mean (SD)	%/ Mean (SD)	%/ Mean (SD)	%/ Mean (SD)	%/ Mean (SD)	%/ Mean (SD)	%/ Mean (SD)	%/ Mean (SD)
**Assessment (diagnostics and routine test)**								
Inspected conjunctiva or examined the client for pallor	12.9	10.1	98.8	100	50	63.3	26	21.3
Asked about, performed, or referred the client for hemoglobin testing	48.2	58.4	7.5	5.1	36.7	46.7	51	58.3
Take the blood pressure	73.3	69.9	98.8	92.3	80	90	38.5	51.9
Examine hand or feet or leg for edema	7.9	6.7	91.3	96.2	26.7	43.3	8.7	8.3
Urine test for albumin or glucose	47.1	56.6	12.5	7.7	36.7	53.3	12.5	7.4
Weight measurement	95.1	78.4	97.5	100	96.7	100	71.2	77.8
Height measurement	5	9.1	67.5	70.5	23.3	20	1	0
Assessment score[1]	**4.1 (2)**	**4.1 (2.3)**	**6.8 (1.3)**	**6.7 (0.9)**	**5 (2.7)**	**6 (2.2)**	**3 (2)**	**3.2 (2.1)**
**Provision and counseling of deworming medicines**								
Explained the purpose of deworming medicines			6.3	5.1	0	30		
Checked for the last dose of deworming tablet taken			6.3	3.8	0	30		
Prescribed or provided albendazole or mebendazole			6.3	6.4	0	16.7	4.8	1.9
Provision and counseling of deworming medicines score[1]			**0.6 (2.2)**	**0.5 (2.1)**	**0 (0)**	**2.6 (4)**	***0.5 (2.1)***	***0.2 (1.4)***
**Provision and counseling for malaria**								
Checked for last dose of malaria prophylactic taken			61.3	47.4	0	23.3		
Explained the purpose of anti-malaria medicine			76.3	65.4	0	26.7		
Gave or prescribed malaria prophylactic			75	78.2	0	16.7		
Explained how to take anti-malaria medicine			78.8	73.1	0	23.3		
Explained side effects of anti-malaria medicine			33.8	20.5	3.3	16.7		
Asked whether PW has mosquito net			57.5	61.5	10	30		
Explained importance of sleeping under malaria net			55	60.3	13.3	23.3		
Gave or instructed PW to obtain mosquito net			42.5	50	0	20		
Provision and counseling malaria score[1]			**6 (3.2)**	**5.7 (2.8)**	**0.3 (0.8)**	**2.3 (3.6)**		

	BANGLADESH		BURKINA FASO		ETHIOPIA		INDIA	
	Intervention	Control	Intervention	Control	Intervention	Control	Intervention	Control
**IFA provision and counseling**								
Prescribed or provided or purchase IFA	90.3	81.2	100	97.4	86.7	86.7	77.9	72.2
Advised on potential side effects of IFA								
Discussed side effects which may occur	20.1	1.6	56.3	29.5	60	50	26.9	15.7
How to manage any IFA side effects/ Increase intake of fruits and vegetables to avoid constipation	42.8	7.5	58.8	30.8	63.3	60	20.2	13.9
Explained how to take IFA								
Advised PW how to take IFA in general			97.5	92.3	90	73.3		
Advised PW to take IFA regularly (1 tablet/day)	86.9	78.8	98.8	100	66.7	70	72.1	79.6
Take at least 180 IFA tablets during pregnancy	28.6	17.2	70	17.9	63.3	43.3		
Continue to take 1 IFA tablet/day up to 3 months after delivery (BD)/ until 42 days postpartum (BF)	28.3	9.9	90	48.7				
Reminded woman not to take IFA with tea or coffee or milk	45.8	2	22.5	7.7	63.3	60	30.8	18.5
Advised to take IFA at nighttime with water or lemon water	59	15.4	31.3	19.2	80	66.7	59.6	59.3
Do not take IFA and calcium tablets together	52.6	12.1					31.7	21.3
Explained any benefit of IFA	51.1	16.6	97.5	82.1	100	83.3	38.5	15.7
IFA provision and counseling score[1]	**5.1 (2.6)**	**2.4 (1.7)**	**7.2 (1.9)**	**5.3 (2)**	**7.5 (2)**	**6.6 (3.3)**	**4.5 (3.1)**	**3.7 (2.6)**
**Calcium provision and counseling**								
Prescribed or provided or purchased calcium	77.4	70.7					76	61.1
Advised on potential side effects of taking calcium								
Discussed side effects which may occur	11.4	2					NA	
How to manage any calcium side effects	11.7	1.4					17.3	6.5
Explained how to take calcium								
Take 2 calcium tablets daily during pregnancy	76.5	53.3					54.8	40.7
Take the first calcium tablet after breakfast	49.9	16.4					50	27.8
Do not take calcium tablets on an empty stomach	45.4	16.8					NA	
Do not take IFA and calcium tablets together	50	11.1					31.7	21.3
Explained any benefit of calcium	46.9	10.3					30.8	11.1
Calcium provision and counseling score[1]	**4.6 (2.9)**	**2.3 (1.9)**					**4.3 (3.4)**	**2.8 (2.9)**
**Diet counseling**								

	BANGLADESH		BURKINA FASO		ETHIOPIA		INDIA	
	Intervention	Control	Intervention	Control	Intervention	Control	Intervention	Control
Healthy nutrition is important for a safe delivery			40	16.7	80	80		
Importance/ benefits of diverse diet	83.8	51.9	80	38.5			52.9	39.8
Advised women consuming at least 5 recommended food groups in a day	80.6	46.1	70	34.6	93.3	66.7	76.9	59.3
Consume fish/meat/ egg daily	85.3	77.4	90	50	90	53.3		
Consume milk/milk products daily	82.6	71.9	83.8	41	86.7	63.3		
Consume dark green leafy vegetables daily	84.9	70.1	95	57.7	90	46.7		
Consume yellow/orange fruit and vegetables daily	81.7	54.1	86.3	52.6	83.3	33.3		
Consume thick daal everyday (BD)/ eat pulses daily (BF)/ legumes daily (ET)	78.8	26.9	67.5	28.2	90	46.7		
Asked about different food items that she has taken in last 24 hours and helped to add the missing food item using locally available nutritious foods			71.3	34.6	83.3	53.3		
Counselled on recommended quantity of food/ increase diet trimester wise	66.7	47.3	61.3	30.8	90	63.3	33.7	19.4
Take nutritious snacks 2 times/day	74	30.7			86.7	66.7		
Diet counseling score[1]	**8 (3.4)**	**5.3 (3.3)**	**7.5 (2.1)**	**3.8 (3.5)**	**8.7 (2.6)**	**5.7 (3.5)**	**5.4 (3.6)**	**4 (3.4)**
**Weight monitoring and weight gain counseling**								
Counseled on the importance of weight gain during pregnancy	61.3	17.8	68.8	15.4	83.3	63.3	60.6	40.7
Explained the weight a PW should gain during pregnancy	75.3	21.8	83.8	43.6	80	53.3	28.8	14.8
Counseled on the importance of taking weight each month to track weight gain			55	11.5	63.3	43.3		
Pregnant women should measure weight every month	63.5	38.4	72.5	41	73.3	63.3		
Weight monitoring and weight gain counseling score[1]	**6.7 (3.8)**	**2.6 (3.2)**	**7 (3.4)**	**2.8 (3.2)**	**7.5 (3.8)**	**5.6 (4.1)**	**4.5 (4.1)**	**2.8 (3.6)**
**Breastfeeding counseling**								
Discussed early initiation of breastfeeding								
Discussed that newborn should be immediately put on skin-to-skin contact	10	1.6	63.7	33.3	33.3	33.3	6.7	4.6
Started breastfeeding immediately after birth	12.9	2.8	87.5	46.2	53.3	36.7	6.7	4.6
Discussed the special properties of colostrum and reasons why it is important	12.1	1.8	83.8	42.3	60	36.7	7.7	5.6

	BANGLADESH		BURKINA FASO		ETHIOPIA		INDIA	
	Intervention	Control	Intervention	Control	Intervention	Control	Intervention	Control
Discussed that newborn should not be given any pre-lacteals like janam ghutti, honey, animal milk etc.	9.7	0.8	60	34.6	53.3	36.7	7.7	3.7
Discussed exclusive breastfeeding								
Counselled to practice exclusive breastfeeding for 6 months	8.3	1.2	58.8	20.5	60	43.3	12.5	7.4
Feed expressed breastmilk if the mother goes out for a long time	1.7	0	15	3.8				
Breastfeeding counseling score[1]	**0.9 (2.4)**	**0.1 (0.9)**	**6.1 (3.2)**	**3 (3.4)**	**5.2 (4.3)**	**3.7 (4.7)**	**0.8 (2.4)**	**0.5 (1.8)**
**Rest and physical activity counseling**								
Advised PW to take rest for at least 2 hours during daytime	84	48.1			50	23.3	49	31.5
Taking rest is important for the growth of the baby	56.8	23.2	33.8	23.1	70	73.3		
Taking rest improves weight gain of the mother	43.6	11.7	31.3	12.8	50	53.3		
A PW should sleep for at least 8 hours a night	81	37.4	18.8	7.7	26.7	36.7		
Avoid heavy work	86.4	56.4	66.3	47.4	86.7	76.7	54.8	37
Rest and physical activity counseling score[1]	**7 (3.2)**	**3.5 (3.1)**	**3.8 (3.6)**	**2.3 (2.9)**	**5.7 (3.2)**	**5.3 (3.3)**	**5.2 (4.6)**	**3.4 (4.3)**

[1]Possible range: 0 – 10

Note: PW- pregnant woman, IFA- Iron and folic acid, BD- Bangladesh, BF- Burkina Faso, ET- Ethiopia

Table S4: Experience of care components in Bangladesh, Burkina Faso, Ethiopia, and India

	BANGLADESH		BURKINA FASO		ETHIOPIA		INDIA	
	Intervention	Control	Intervention	Control	Intervention	Control	Intervention	Control
	%/ Mean (SD) N=720	%/ Mean (SD) N=495	%/ Mean (SD) N=80	%/ Mean (SD) N=78	%/ Mean (SD) N=30	%/ Mean (SD) N=30	%/ Mean (SD) N= 104	%/ Mean (SD) N= 108
**Client satisfaction**								
Behavior of facility employees	97.6	97.6	97.5	97.4	86.7	90		
Interaction with antenatal care provider	98.8	97.8	98.8	97.4	90	90		
Waiting time	91.7	94.9	91.3	96.2	86.7	76.7		
Waiting area	96.9	98.2	95	93.6	86.2	80		
Facility hours of operation	98.6	98.6	97.5	93.6	86.7	73.3		
Cleanliness of facility	98.2	98.6	98.8	94.9	93.3	76.7		
Overall experience	97.1	96.4	97.5	98.7	90	93.3		
Client satisfaction score[1]	**9.7 (0.8)**	**9.7 (1)**	**9.7 (0.9)**	**9.6 (1.2)**	**8.8 (2.7)**	**8.3 (3)**		
**Client's experience**								
Greeted the woman/family with respect	99.7	97.6	97.5	98.7	86.7	86.7	78.8	85.2
Gives only relevant suggestions	87.5	84	71.3	61.5	76.7	83.3		
Reflects back what the beneficiary says	70.6	48.7	86.3	84.6	50	56.7		
Maintains eye contact with the beneficiary	99.7	96.2	96.3	100	86.7	100		
Uses non-verbal communication	71.4	69.1	96.3	91	66.7	66.7		
Asks open-ended questions	87.4	73.9	97.5	96.2	60	56.7		
Speaks slowly and clearly	99.6	97.2	98.8	100	90	100	61.5	63.9
Uses simple, easy-to-understand language	95.7	93.5	97.5	98.7	93.3	93.3		
Asked the woman if she had any questions and encouraged questions	45.6	18.2	83.8	73.1	70	73.3		
Listened carefully to PW's concerns or questions	100	98	97.5	100	93.3	96.7	53.8	58.3
Did not use judgmental words	37.4	66.3	95	88.5	50	53.3	35.6	37
Praises things that the beneficiary is doing right	48.9	7.9	77.5	64.1	63.3	60	28.8	21.3
Used IEC material for counselling	74.4	1.2	86.3	60.3	83.3	33.3	33.7	6.5
Health providers schedule the next ANC visit			100	100	80	90		
Plan to come back for the next checkup	98.8	97	98.8	98.7	100	100		
Will recommend to others	97.8	96.6	100	98.7	93.3	100		
Client's experience score[1]	**8.2 (1.1)**	**7.1 (1.1)**	**9.3 (1.3)**	**8.9 (1.1)**	**7.8 (2.1)**	**7.9 (1)**	**4.9 (2.5)**	**4.5 (2.1)**

[1]Possible range: 0 - 10

Note: IEC- Information education and communication, ANC- Antenatal care
